# Prevalence of self-reported mental disorders in pregnancy and associations with adverse neonatal outcomes: a population-based cross-sectional study

**DOI:** 10.1186/s12884-019-2572-4

**Published:** 2019-11-08

**Authors:** David Mongan, Janine Lynch, Donncha Hanna, Ciaran Shannon, Shona Hamilton, Claire Potter, Colin Gorman, Orlagh McCambridge, Rachel Morrow, Ciaran Mulholland

**Affiliations:** 1 School of Medicine, Queen’s University Belfast, University Road, Belfast, BT7 1NN Northern Ireland; 2 RCSI Education and Research Centre, Beaumont Hospital, Dublin 9, Ireland; 3 Belfast Health and Social Care Trust, Trust Headquarters, A Floor, Belfast City Hospital, Lisburn Road, Belfast, County Antrim BT9 7AB Northern Ireland; 4 School of Psychology, Queen’s University Belfast, University Road, Belfast, BT7 1NN Northern Ireland; 5 Northern Health and Social Care Trust, Trust Headquarters, Bretten Hall, Bush Road, Antrim, County Antrim BT41 2RL Northern Ireland; 6 Southern Health and Social Care Trust, Trust Headquarters, Bretten Hall, Bush Road, Antrim, County Antrim BT41 2RL Northern Ireland

**Keywords:** Mental disorder, Perinatal mental health, Pregnancy, Adverse neonatal outcomes, Perinatal depression, Postnatal psychosis

## Abstract

**Background:**

Mental disorders in pregnancy are common causes of morbidity and mortality with associated risks of adverse neonatal outcomes. Our aims were to evaluate the prevalence of self-reported mental disorders in women presenting to maternity services and to determine the association between history of self-reported maternal mental disorder and adverse neonatal outcomes.

**Methods:**

Data on all singleton pregnancies known to maternity services in Northern Ireland over the period 2010 to 2015 were extracted from the Northern Ireland Maternity System (NIMATS), including frequency data for number of pregnancies where the mother reported a history of mental disorder. Odds ratios were derived from logistic regression analyses to determine the associations between self-reported maternal mental disorder and preterm birth, low infant birth weight and APGAR scores.

**Results:**

In total, 140,569 singleton pregnancies were registered using NIMATS over this period. In 18.9% of these pregnancies, the mother reported a history of at least one mental disorder. After adjustment for potential confounding factors, significant associations were demonstrated between self-reported maternal mental disorder and preterm birth (odds ratio [OR] 1.31, 95% confidence interval [CI] 1.25–1.37), low infant birth weight (OR 1.29, 95% CI 1.21–1.38) and APGAR score < 7 at 1 min (OR 1.14, 95% CI 1.10–1.19) and 5 min (OR 1.23, 95% CI 1.12 to 1.34).

**Conclusions:**

These findings emphasise the critical importance of routine enquiry regarding psychiatric history when women present to maternity services and the impact of maternal mental illnesses upon outcomes for their infants.

## Background

Mental disorders are common causes of morbidity and mortality for women in the perinatal period. In the UK and Ireland, from 2009 to 2013 there were 3.7 deaths per 100,000 pregnancies from mental health-related causes during or up to 1 year after the end of pregnancy; 101 women died by suicide over the same period, representing one in seven of all maternal deaths [1]. Quite aside from the severe human cost, the economic burden is also considerable; perinatal depression, anxiety and psychosis carry an estimated long-term cost to society of approximately £8.1 billion for each one-year cohort of births in the UK [2]. Antenatal identification of women with a history of mental illness is important to protect the health of the mother, but may also impact upon the future health of the neonate.

### Effects of pregnancy on maternal mental health

Women with a history of severe mental illness may experience relapse or deterioration of their condition in the perinatal period. For example, a prospective investigation found 43% of a cohort of women with a history of major depression relapsed during their pregnancy [3]. In bipolar disorder, risk of pregnancy-associated relapse may be as high as 50% [4]. A specific association exists between bipolar disorder and postpartum psychosis, a severe psychiatric condition with associated risks to the life of the mother and, potentially, her baby if not recognised and managed appropriately. In women with bipolar disorder and a personal or family history of postnatal psychosis the risk increases to approximately 60% [5], although, as for other perinatal mental illnesses, the disorder may also arise de novo. For women with schizophrenia, the postpartum period in particular can be associated with a risk of psychotic relapse [6] for which careful monitoring is required. Other conditions associated with a risk of perinatal relapse include anxiety disorders such as generalised anxiety disorder and obsessive-compulsive disorder [7] as well as eating disorders [8]. Mental disorders frequently co-occur with other problems and stressors in pregnancy such as substance misuse [9] and intimate partner violence (IPV) [10] which can further increase the risk of relapse.

### Effects of maternal mental disorder on neonatal outcomes

In addition to the impact of pregnancy upon a mother’s mental health (whether in the context of a pre-existing mental disorder or otherwise), mental disorders in pregnancy have been associated with adverse neonatal outcomes in previous studies. Results of two meta-analyses indicate an association between maternal depression and preterm birth, although conflicting results were found for low birth weight [11, 12]. Maternal bipolar disorder has been associated with small-for-gestational-age births [13]. Schizophrenia has been associated with intra-uterine growth restriction, reduced APGAR scores at birth and congenital abnormalities [6] as well as low birth weight [14]. Women with eating disorders are also at risk of delivering low birth weight infants [15]. In a population-based study, self-reported anxiety disorders were associated with low APGAR score, although not with low birth weight or preterm birth [16].

### Factors influencing the association between maternal mental disorder and adverse neonatal outcomes

Several factors may influence observed associations between maternal mental disorder and adverse neonatal outcomes. Smoking is more common in people with mental health problems [17] and is associated with adverse neonatal outcomes such as intrauterine growth retardation and placental complications [18]. Alcohol use disorders are frequently co-morbid with other mental illnesses, particularly mood and anxiety disorders [19]. A population-based prospective cohort study found associations between drinking one or more alcoholic drink per day during pregnancy and outcomes of low birth weight and preterm birth [20]. Maternal age is also relevant as women aged > 35 years appear to be at increased risk of preterm birth as well as low birth weight [21]. Pregnancy in adolescence has been associated with risk of premature birth [22] and young mothers may be particularly vulnerable for poor mental health outcomes [23]. Risk of obesity and cardiovascular diseases such as hypertension is increased in people with severe mental illness [24]. This likely relates to an interplay of multiple psychosocial factors as well as the effects of psychotropic medications [25]. In pregnancy, obesity [26] and hypertensive disorders such as chronic hypertension [27] and pre-eclampsia [28] have been associated with adverse neonatal outcomes. Preterm birth and low birth weight have also been associated with low maternal BMI [29, 30] which may occur in eating disorders such as anorexia nervosa. Past or active eating disorders in pregnancy have been associated with low birth weight [15]. Fetal abnormalities in pregnancy have a range of possible contributory causes. In the context of maternal mental disorders, fetal abnormalities may be related in part to use of prescribed [31] and illicit drugs [32] with potential teratogenic effects. Social factors are also pertinent; for example, low socioeconomic status [33] and lone parenting [34] have been linked epidemiologically with low birth weight, and are also associated with mental disorders [35, 36]. Experience of IPV is more common in mothers with mental illness compared to those without mental illness [37] and is in itself associated with adverse outcomes for the neonate [38, 39].

### Study aims

Despite the recognised importance of mental illness in pregnancy, limited data exist regarding the proportion of women presenting to maternity services who have a history of mental disorder. The aims of the present study were two-fold. Firstly, we aimed to determine the prevalence of self-reported severe mental disorders in women presenting to maternity services in Northern Ireland. Secondly, we aimed to assess the relationship between self-reported history of maternal mental disorder and preterm birth, low infant birth weight and low APGAR scores, after adjusting for a range of potential confounding variables. We hypothesised that maternal history of mental disorder would be associated with adverse neonatal outcomes.

## Methods

### Study design

This study took the form of a cross-sectional database analysis using data derived from the Northern Ireland Maternity System (NIMATS), an electronic database used by midwives throughout Northern Ireland to record routine clinical data for all women presenting to maternity services. As well as a variety of antenatal, perinatal and postnatal information, all mothers are screened for a history of mental disorders by interview with a midwife at the booking appointment in accordance with UK guidelines [40]. Midwives ask women if they have a history of the following disorders: schizophrenia; bipolar disorder; severe obsessive-compulsive disorder; severe eating disorder; severe depression; and postpartum psychosis. Midwives rely upon women self-reporting and/or information from their general practitioner via the referral to maternity services.

### Data extraction

Data were obtained from NIMATS for all women presenting to maternity services in Northern Ireland during the period January 2010 – December 2015. All antenatal and neonatal data were recorded in the same database. Data extraction was performed in collaboration with the Northern Ireland Health and Social Care Honest Broker Service (HBS). All HBS processes are in line with data protection principles, confidentiality requirements and the Information Commissioner’s Office codes of practice. HBS provides a safe and secure environment in which data are processed. The dataset was anonymised before provision to the research team in a ‘safe haven’ setting and all outputs were subject to statistical disclosure control. Researchers who directly accessed the dataset completed Safe User of Research data Environments (SURE) accredited training.

Frequency data were obtained for the number of pregnancies where the mother reported a history of one or more of the following mental disorders on routine screening by midwives at their booking appointment: schizophrenia; bipolar disorder; severe obsessive-compulsive disorder; severe eating disorder; severe depression; and postpartum psychosis. Mental disorders aside from these are recorded in the database as ‘other mental disorder’. Data were also collected regarding antenatal factors including the mothers’ age and body mass index (BMI) at the booking appointment; use of alcohol; smoking status; employment status of the mother; parenting status of the mother (lone parent or not); disclosure of IPV on routine enquiry; presence of fetal abnormality; and presence of hypertensive disorder in pregnancy. Data for antenatal smoking status was based on maternal self-report as either current smoker or current non-smoker. Alcohol status was also based on maternal self-report as either current drinker or non-drinker. Neonatal outcome data included gestational age at birth; infant birth weight; and APGAR score at 1 and 5 min postpartum.

### Statistical analysis

Statistical analysis was performed in SPSS Statistics software version 24 (Armonk, NY: IBM Corp). Logistic regression was used to derive odds ratios for associations between the exposure (self-reported history of at least one mental disorder at the booking appointment) and the following outcomes: low birth weight (defined according to WHO criteria as < 2500 g); preterm birth (defined according to WHO criteria as gestational age < 37 weeks at delivery); and APGAR score < 7 at 1 and 5 min (APGAR score < 7 indicates that the neonate requires medical attention). Analyses were performed on the basis of the mother reporting a history of at least one mental disorder (rather than for each disorder individually), producing a dichotomous exposure variable (history of mental disorder/no history of mental disorder). The assumption of non-multicollinearity was satisfied by inspection of variance inflation factors [41]. We excluded pregnancies with multiple births due to the known association between multiple births and adverse perinatal outcomes [42]. Pregnancies where at least one variable value was missing were excluded from the regression analyses.

### Covariates

For adjusted analyses, covariates were chosen based on prior associations with mental disorder and neonatal outcomes. These included: self-reported antenatal smoking status at booking appointment (smoker/non-smoker); self-reported antenatal alcohol status at booking appointment (drinker/non-drinker); maternal age at booking < 18 or > 35 years (with reference to maternal age 18–35 years); maternal BMI at booking < 18.5 kg/m^2^ or > 25 kg/m^2^ (with reference to maternal BMI 18.5–25 kg/m^2^); whether or not the mother reported a previously-undisclosed experience of IPV on routine enquiry at booking (new disclosure/no new disclosure); employment status of mother (employed/unemployed); parenting status (lone parent/not lone parent); fetal abnormality registered during pregnancy (present/not present); and hypertensive disorder (pre-eclampsia, chronic hypertension or gestational hypertension) registered during pregnancy (present/not present). For the outcome of APGAR score < 7, preterm birth and low birth weight were included as additional covariates.

## Results

During the period January 2010 – December 2015 there were 142,772 pregnancies registered using NIMATS. Following exclusion of pregnancies with multiple births, the final analysed dataset comprised 140,569 pregnancies.

Table 1 presents descriptive data for the major antenatal variables examined, including: self-reported history of mental disorder; family history of bipolar disorder or postpartum psychosis; alcohol and smoking status at booking; and antenatal disclosure of IPV. In 26,547 pregnancies (18.87%) the mother reported a history of at least one mental disorder. The majority of these were recorded as ‘other mental disorder’. Table 2 presents descriptive data for the adverse neonatal outcomes examined: low birth weight; preterm birth; and APGAR score < 7 at 1 and 5 min.

**Table 1:** Frequency data for self-reported history of mental disorder, family history of bipolar disorder or postnatal psychosis, alcohol and smoking status at booking and disclosure of intimate partner violence for singleton pregnancies in 2010–2015 (*n* = 140,569)

	Frequency	% of total
History of mental disorder		
Schizophrenia	49	0.03
Bipolar disorder	212	0.15
Postpartum psychosis	483	0.34
Severe depression	7549	5.37
Severe eating disorder	793	0.56
Severe obsessive-compulsive disorder	334	0.24
Other mental disorder	17,127	12.18
Any mental disorder	26,547	18.87
Family history of mental disorder		
Bipolar disorder	2238	1.59
Postnatal psychosis	709	0.50
Alcohol status at booking		
Non-drinker	139,318	99.11
Drinker	1141	0.81
Not recorded	110	0.08
Smoking status at booking		
Non-smoker	118,168	84.06
Smoker	22,306	15.87
Not recorded	95	0.07
Antenatal disclosure of intimate partner violence		
No disclosure	131,080	93.25
Historical disclosure	3188	2.27
New disclosure, family receiving support	789	0.56
New disclosure, risk assessment and referral to appropriate services	373	0.27
Not recorded	5139	3.65

**Table 2:** Frequency data for birth weight, gestational age at birth and APGAR score at 1 and 5 min for singleton pregnancies in 2010–2015 (*n* = 140,569)

	Frequency	% of total
Birth weight		
Low birth weight (< 2500 g)	6451	4.59
Normal birth weight (2500 – 3999 g)	112,981	80.37
High birth weight (> 4000 g)	20,949	14.90
Not recorded	188	0.13
Gestational age at birth		
< 37 weeks	15,984	11.37
≥ 37 weeks	12,4579	88.62
Not recorded	6	0.01
APGAR score at 1 min		
Score < 7	10,604	7.54
Score 7–10	129,470	92.10
Not recorded	495	0.35
APGAR score at 5 min		
Score < 7	2191	1.56
Score 7–10	137,793	98.03
Not recorded	585	0.42

For unadjusted analyses, complete case data were available for 99% of the dataset. Unadjusted analyses (Table 3) provided evidence for associations between self-reported mental disorder and preterm birth (odds ratio [OR] 1.43, 95% confidence interval [CI] 1.37–1.48, *p* < 0.001), low infant birth weight (OR 1.58, 95% CI 1.49–1.68, *p* < 0.001), and APGAR score < 7 at 1 min (OR 1.22, 95% CI 1.17–1.27, *p* < 0.001) and at 5 min (OR 1.42, 95% CI 1.31–1.54, *p* < 0.001).

**Table 3:** Univariate logistic regression analyses for associations between self-reported mental disorder and preterm birth, low infant birth weight and APGAR score < 7 at 1 and 5 min for singleton pregnancies in 2010–2015 (*n* = 140,569)

	Beta	SE	Wald	*P*	Odds ratio	Lower 95% CI	Upper 95% CI	Missing cases (%)
Preterm birth	0.35	0.02	304.86	< 0.001	1.43	1.37	1.48	< 0.1
Low infant birth weight	0.46	0.03	240.44	< 0.001	1.58	1.49	1.68	0.1
APGAR score < 7 at 1 min	0.20	0.02	97.15	< 0.001	1.22	1.17	1.27	0.4
APGAR score < 7 at 5 min	0.35	0.04	74.90	< 0.001	1.42	1.31	1.54	0.4

*SE* Standard error, *CI* Confidence interval

For adjusted analyses, complete case data were available for approximately 95% of the dataset. Adjusted odds ratios (aOR) for self-reported mental disorder and adverse neonatal outcomes were of diminished magnitude, but remained significant (Table 4): for preterm birth, aOR: 1.31 (95% CI 1.25–1.37, *p* < 0.001); for low infant birth weight, aOR: 1.29 (95% CI 1.21–1.38, *p* < 0.001); for APGAR score < 7 at 1 min aOR: 1.14 (95% CI 1.10–1.19, *p* < 0.001); and for APGAR score < 7 at 5 min aOR: 1.23 (95% CI 1.12–1.34, *p* < 0.001).

**Table 4:** Multivariable logistic regression analyses for preterm birth, low infant birth weight and APGAR score < 7 at 1 and 5 min for singleton pregnancies in 2010–2015 (*n* = 140,569)

	Beta	SE	Wald	*P*	Odds ratio	Lower 95% CI	Upper 95% CI
1) Preterm birth Missing cases: 4.8%							
Age < 18	− 0.17	0.07	5.14	0.023	0.85	0.74	0.98
Age > 35	0.32	0.02	177.67	< 0.001	1.38	1.32	1.45
BMI < 18.5	0.44	0.05	66.35	< 0.001	1.55	1.39	1.72
BMI > 25	0.00	0.02	0.00	0.978	1.00	0.97	1.04
Current alcohol use	−0.09	0.10	0.87	0.352	0.91	0.76	1.10
Current smoking	0.36	0.02	244.97	< 0.001	1.44	1.38	1.51
Fetal abnormality in pregnancy	1.93	0.08	532.92	< 0.001	6.89	5.85	8.12
Hypertensive disorder in pregnancy	1.55	0.04	1487.49	< 0.001	4.69	4.34	5.08
Lone parent	0.05	0.04	1.83	0.176	1.05	0.98	1.12
Maternal employment	−0.19	0.02	81.18	< 0.001	0.83	0.80	0.87
New antenatal disclosure of intimate partner violence	0.32	0.22	2.08	0.149	1.38	0.89	2.12
Self-reported mental disorder	0.27	0.02	151.92	< 0.001	1.31	1.25	1.37
2) Low infant birth weight Missing cases: 4.9%							
Age < 18	−0.15	0.10	2.15	0.143	0.87	0.71	1.05
Age > 35	0.31	0.04	64.48	< 0.001	1.36	1.26	1.47
BMI < 18.5	0.73	0.07	116.80	< 0.001	2.07	1.81	2.36
BMI > 25	−0.28	0.03	100.11	< 0.001	0.76	0.71	0.80
Current alcohol use	0.03	0.13	0.06	0.811	1.03	0.80	1.33
Current smoking	0.93	0.03	845.05	< 0.001	2.54	2.39	2.71
Fetal abnormality in pregnancy	2.67	0.09	891.55	< 0.001	14.49	12.16	17.27
Hypertensive disorder in pregnancy	2.03	0.05	1714.25	< 0.001	7.64	6.94	8.41
Lone parent	0.22	0.05	21.08	< 0.001	1.24	1.13	1.36
Maternal employment	−0.22	0.03	48.44	< 0.001	0.81	0.76	0.86
New antenatal disclosure of intimate partner violence	0.62	0.26	5.66	0.017	1.86	1.12	3.12
Self-reported mental disorder	0.26	0.03	62.04	< 0.001	1.29	1.21	1.38
3) APGAR score < 7 at 1 min Missing cases: 5.2%							
Age < 18	0.27	0.06	17.78	< 0.001	1.31	1.16	1.49
Age > 35	−0.01	0.03	0.26	0.609	0.99	0.94	1.04
BMI < 18.5	−0.10	0.06	2.72	0.099	0.90	0.80	1.02
BMI > 25	0.19	0.02	127.74	< 0.001	1.21	1.17	1.26
Current alcohol use	−0.02	0.10	0.03	0.861	0.98	0.82	1.18
Current smoking	−0.05	0.02	4.58	0.032	0.95	0.91	1.00
Fetal abnormality in pregnancy	1.63	0.09	334.30	< 0.001	5.09	4.27	6.06
Hypertensive disorder in pregnancy	0.34	0.05	51.73	< 0.001	1.41	1.28	1.55
Lone parent	0.22	0.03	43.10	< 0.001	1.24	1.16	1.32
Low infant birth weight	1.08	0.04	905.38	< 0.001	2.93	2.73	3.15
Maternal employment	0.03	0.02	2.25	0.133	1.03	0.99	1.07
New antenatal disclosure of intimate partner violence	−0.07	0.25	0.08	0.781	0.93	0.57	1.52
Preterm birth	0.42	0.03	236.12	< 0.001	1.52	1.44	1.60
Self-reported mental disorder	0.13	0.02	37.44	< 0.001	1.14	1.10	1.19
4) APGAR score < 7 at 5 min Missing cases: 5.2%							
Age < 18	−0.06	0.15	0.17	0.685	0.94	0.70	1.26
Age > 35	−0.05	0.05	0.73	0.392	0.96	0.86	1.06
BMI < 18.5	−0.01	0.12	0.01	0.919	0.99	0.78	1.25
BMI > 25	0.18	0.04	22.19	< 0.001	1.19	1.11	1.28
Current alcohol use	0.22	0.18	1.45	0.228	1.24	0.87	1.78
Current smoking	−0.04	0.05	0.64	0.426	0.96	0.87	1.06
Fetal abnormality in pregnancy	2.41	0.10	556.73	< 0.001	11.15	9.13	13.62
Hypertensive disorder in pregnancy	−0.02	0.09	0.04	0.840	0.98	0.82	1.18
Lone parent	0.23	0.07	11.19	0.001	1.25	1.10	1.43
Low infant birth weight	1.50	0.06	688.59	< 0.001	4.48	4.00	5.01
Maternal employment	0.04	0.04	0.98	0.323	1.04	0.96	1.14
New antenatal disclosure of intimate partner violence	−0.04	0.47	0.01	0.941	0.97	0.38	2.44
Preterm birth	0.95	0.05	355.79	< 0.001	2.59	2.34	2.85
Self-reported mental disorder	0.21	0.05	20.61	< 0.001	1.23	1.12	1.34

*SE* Standard error, *CI* Confidence interval

## Discussion

This study highlights that in a significant proportion (almost one-fifth) of pregnancies in Northern Ireland, the mother reports a history of mental disorder. The most recent National Institute for Health and Care Excellence guidelines for perinatal mental healthcare state that women with a history of severe mental illness should be referred to a secondary mental health service, and preferably a specialist perinatal mental health service [40]. Despite this, service provision is widely variable across the UK and Ireland. In Northern Ireland, 80% of women do not have access to specialist perinatal mental health services [2]. Considered alongside evidence that psychiatric mother and baby units have established clinical benefits [43] and are cost-effective [40] our data underline the need for further development of such services.

In line with previous evidence, we found small but significant associations between self-reported history of mental disorder and preterm birth, low infant birth weight and APGAR score < 7. Our findings reinforce the importance of routine enquiry regarding history of mental health problems in women presenting to maternity services. In Northern Ireland, routine enquiry exists for six severe mental illnesses. Most self-reported disorders were recorded as ‘other mental disorder’. This category may include women with other common mental disorders such as anxiety disorders and post-traumatic stress disorder. Anxiety and stress during pregnancy may be associated with later adverse developmental effects for the child [44, 45]. Subclinical (but nevertheless significant) prenatal stress may thus warrant inclusion in antenatal screening and monitoring, in addition to the severe mental illnesses currently included in routine enquiry.

In adjusted analyses, the associations between maternal mental disorder and neonatal outcomes persisted but attenuated, suggesting the presence of confounding by some of the included covariates. While not the focus of this study, particularly strong associations with adverse outcomes were observed for fetal abnormality and hypertensive disorders in pregnancy, in line with previous research [27, 28, 46, 47]. In our study, associations with preterm birth were also found for young and advanced maternal age, low BMI, smoking and employment status. Low birth weight was additionally associated with new antenatal disclosure of IPV, high BMI and being a lone parent. Low APGAR score at 5 min was associated with high BMI, being a lone parent, fetal abnormality, preterm birth and low infant birth weight as well as maternal mental disorder. Previous studies have reported adverse outcomes in association with maternal smoking [48, 49], BMI [50] and advanced maternal age [51]. The observed associations with social factors such as lone parenting and employment status are consistent with previous research investigating the influence of socio-economic factors upon birth outcomes [52, 53]. We found antenatal disclosure of IPV was associated with low birth weight though not preterm birth, despite previous evidence that this exposure is associated with both outcomes [54, 55]. However the reported prevalence in our study is small, and may be an under-estimate of the actual prevalence [56, 57].

The exposure in our study was self-reported history of mental disorder at the booking appointment. We did not have access to data regarding disorders arising de novo in the antenatal period and thus were unable to assess the association of such disorders on neonatal outcomes. Anxiety during pregnancy has been associated with preterm birth and low birth weight in meta-analysis [58]. There is also evidence for an association between antepartum depression and outcomes of preterm birth and low birth weight, with stronger effects for preterm birth in developing compared to developed countries [11]. Risk of low birth weight is increased in mothers with a history of schizophrenia, but the risk is highest for mothers who experience relapse of their illness during pregnancy [59] suggesting that the timing of acute episodes does affect risk of adverse outcomes. Nevertheless, history of disorder is an important risk indicator for experience of disorder during pregnancy [60, 61] and thus screening for this exposure facilitates identification of mothers at greater risk of mental health problems in the perinatal period.

There are several possible reasons to explain the relationship between maternal mental disorder and adverse neonatal outcomes. Women living with mental health problems may be unable to engage optimally with antenatal services. As a result, potential problems related to the health of the fetus or the mother may not be identified, or identified at a later stage in the pregnancy. Further, women with a history of mental disorders may be more likely to have a history of other risk factors associated with adverse perinatal outcomes such as substance use [62] or co-morbid medical conditions [63]. Women with mental disorders may also be more likely to experience obstetric complications [64]. For example, associations have been found between depression in late pregnancy and epidural analgesia [65]. Women with a history of schizophrenia have been shown to be at increased risk of pre-eclampsia, placental abruption, gestational diabetes, venous thromboembolism and need for labour induction or Caesarean section [66]. Finally, biological hypotheses have been advanced such as shared genetic risk factors between mother and child, epigenetic changes and gene-environment interactions [67]. The neuroendocrine system, and in particular the hypothalamic-pituitary axis, represents a biological mechanism by which maternal stress in pregnancy could influence the growing fetus; for example, by affecting uterine blood flow [68]. In reality, the mechanisms underlying the association between maternal mental disorder and adverse neonatal outcomes likely involve complex interactions of biological and psychosocial factors which warrant further elucidatory investigation.

This study has several strengths. NIMATS is used routinely on a regional basis and thus we can be confident the data derived from this source are representative of the population. The large sample size affords precision to our estimates of the associations between self-reported maternal mental disorder and adverse neonatal outcomes. Our findings are based on routinely-collected clinical data and are likely generalisable to countries with similar obstetric healthcare systems, although may be less applicable in less economically developed countries [69].

Our study also has several limitations. Firstly, many data obtained from NIMATS (including history of maternal mental disorder) are self-reported. Women may have inaccurately reported the presence, absence, or type of mental disorder and it was not possible to verify the diagnosis from primary or other secondary healthcare records. Furthermore, it is possible that the figures obtained for self-reported mental disorder, use of alcohol, smoking and experience of IPV are under-estimates given the known stigma surrounding these issues, particularly in the context of pregnancy [70, 71]. Most self-reported mental disorders were recorded in the dataset as ‘other disorder’ and it was not possible to sub-categorise by type of disorder for this group. We based our regression analyses on the presence or absence of at least one self-reported disorder and were therefore unable to examine for a differential effect dependent on the specific type of disorder, or for the effect of multiple psychiatric co-morbidities. Approximately 5% of pregnancies were excluded from our adjusted analyses due to missing data. We were unable to adjust for all potential confounders, and in particular, for illicit and prescribed drug use during pregnancy [9, 72]. Lastly, the cross-sectional nature of our analyses implies association but not causation with respect to the relationship between maternal mental disorder and adverse neonatal outcomes.

## Conclusions

The results of this study highlight that a significant proportion of women presenting to maternity services report a history of mental disorder. Routine enquiry regarding a history of mental disorder is particularly important in this population, given that the perinatal period can be associated with relapse of known diagnoses or occurrence of new disorders. Furthermore, our data support associations between maternal mental disorder and adverse neonatal outcomes. Future research may focus on further eliciting the multiple biological, psychological and social aetiological pathways that mediate this association, with a view to implementing preventative strategies which could be integrated into maternal and mental healthcare service provision to improve outcomes for mothers and their infants.

## Data Availability

The datasets generated and/or analysed during the current study are not publicly available because they were generated specifically for the purposes of this research project from Northern Ireland Health and Social Care (HSC) data by the Honest Broker Service (HBS). For further information regarding data availability and how researchers can request access to non-identifiable versions of HSC datasets, please see http://www.hscbusiness.hscni.net/services/2454.htm.

## Abbreviations

aOR: Adjusted odds ratio
APGAR: Appearance/Pulse/Grimace/Activity/Respiration score
BMI: Body mass index
HBS: Honest Broker Service
NIMATS: Northern Ireland Maternity System
OR: Odds ratio
